# Scattering Intensity and Directionality Probed Along Individual Zinc Oxide Nanorods with Precisely Controlled Light Polarization and Nanorod Orientation

**DOI:** 10.3390/photonics2020684

**Published:** 2015-06-18

**Authors:** Daniel S. Choi, Manpreet Singh, Sheng Song, Jae Young Chang, Yongkoo Kang, Jong-in Hahm

**Affiliations:** Department of Chemistry, Georgetown University, 37th & O Sts. NW., Washington, DC 20057, USA

**Keywords:** zinc oxide nanorod, far field scattering, back-aperture imaging, single nanorod scatterer, polarized light interaction, light matter relationship, nanophotonics

## Abstract

We elucidated the light-matter interaction of individual ZnO NRs with a monochromatic beam of linearly polarized light that scatters elastically from the ZnO NRs by performing forward scattering and back-aperture imaging in a dark-field setting. We precisely controlled the electric field vector of the incident light and the NR orientation within the plane of light interaction during both modes of measurement, and spatially resolved the scattering response from different interaction points along the NR long axis. We then discerned, for the first time, the effects of light polarization, analyzer angle, and NR orientation on the intensity and directionality of the optical responses both qualitatively and quantitatively along the length of the single ZnO NRs. We identified distinctive scattering profiles from individual ZnO NRs subject to incident light polarization with controlled NR orientation from the forward dark-field scattering and back-aperture imaging modes. The fundamental light interaction behavior of ZnO NRs is likely to govern their functional outcomes in photonics, optoelectronics, and sensor devices. Hence, our efforts provided much needed insight into unique optical responses from individual 1D ZnO nanomaterials, which could be highly beneficial in developing next-generation optoelectronic systems and optical biodetectors with improved device efficiency and sensitivity.

## 1. Introduction

The optical properties of one-dimensional zinc oxide nanorods (1D ZnO NRs) have been extensively studied and engineered for better photonic [1–7], optoelectronic [8–14], and biosensing applications [15–18]. Many studies have previously demonstrated that the incorporation of 1D ZnO materials in those applications can enable improvements in emission efficiency and/or detection sensitivity over their 2D and bulk counterparts [11,19–25]. From these efforts, beneficial optoelectronic characteristics of 1D ZnO have been identified that have not been readily observed from the thin film or bulk form of ZnO. For instance, single-crystalline ZnO NRs have been shown to exhibit beneficial optical properties such as spontaneous/stimulated emission at room temperature [21,26–30] and effective UV/visible light guiding [2–4,7,24]. In other examples, the optical signal-enhancing capability of ZnO NRs has been used in conjunction with dye-coupled DNA and proteins for fluorescence-based biomedical detection [15–18,25,31–33].

Both individual and ensembles of ZnO NRs have been exploited for the aforementioned applications. Upon subjecting 1D ZnO to incident light with different measurement configurations for excitation and detection, light can trigger various responses from the materials such as bandgap emission and elastic scattering. In this regard, the majority of previous studies on 1D ZnO has focused on ensembles of NRs for a test platform to be investigated, and on the bandgap-associated photoluminescence for a property to be examined. Several studies have attempted to elucidate interesting light behavior specifically from individual ZnO nanomaterials instead of ensembles [2,4,24,34,35]. Examples of these endeavors can be found in the areas of subwavelength waveguiding using 1D structures such as nanoribbons and nanobelts [23,24,29]. Another study in the field of hybrid plasmonic/photonic coupler has demonstrated that a paired system of individual ZnO NRs and metal nanowires can be used to convert light to plasmons via direct light coupling and *vice versa* [6]. Very recently, crystal facet-dependent fluorescence emission characteristics have been reported from dye-conjugated proteins on different crystal surfaces of isolated ZnO NRs [36]. In these investigations employing single ZnO NRs as test elements, light is known to couple effectively into the ZnO NR medium and propagate predominantly along the long axis of the ZnO NR via guided- and/or surface evanescent-waves. This highly directional light guiding and propagation along the c-axis of ZnO NRs occur whether incident light is launched from another nanowire source for the cases of subwavelength waveguiding and hybrid coupling, or whether emitted light from biomolecules is coupled to the high refractive index NR medium instead.

For many optical and bio-optical applications such as those described above, quantifying how individual ZnO NRs scatter light to far field is also critical since far-field observations of the light signal are commonly utilized in many optical detection schemes. Information on scattering intensity and directionality can provide much-needed guiding principles for optimizing optical device outputs and for accurate bio-optical signal interpretation in both single and ensemble ZnO NR-based devices. Therefore, scattering is another important aspect of light response that needs to be thoroughly probed to establish the fundamental light-matter relationship. However, it is not yet clearly understood how light from ZnO NRs scatter into far field. Due to the inherently low signal intensity that can be collected from a single nanomaterial and the difficulty of removing the background optical noise, very few studies have systematically investigated scattering from semiconducting oxide NRs, especially by addressing each NR independently [37,38].

In this article, we study the optical responses of individual ZnO NRs upon illumination with a linearly polarized, monochromatic beam of light (642 nm in wavelength), while specifically focusing on elucidating light polarization- and NR orientation-dependent, elastic scattering characteristics of single ZnO NRs. To circumvent the challenges of low signal and high background issues in single NR imaging, we utilize dark field (DF)-based optical measurement schemes capable of detecting the scattering intensity and, at the same time, recording the spatial distribution from individual NRs upon illumination with a well-defined incident wave vector and polarization. Specifically, we carry out DF scattering measurements in forward geometry as well as back-aperture imaging (also known as Fourier microscopy) in order to quantify systematically the position-, orientation-, and polarization-resolved optical response from individual ZnO NRs. The two investigation modes utilized in this study are designed to provide not only the scattering intensity but also scattering directionality from each NR. In the forward DF scattering mode, the scattering signal from individual ZnO NRs is both qualitatively and quantitatively discerned along the position on the NR. This measurement is systematically conducted as a function of analyzer rotation when the ZnO NRs are illuminated under different electric field orientations of the incident light. We also ascertain distinctive, polarization-dependent scattering responses from single ZnO NRs that are arranged in different directions within the sample plane. In the back-aperture imaging mode, we identify the spatial distribution of the scattering signal from individual ZnO NRs. We resolve the polarization-dependent scattering directivity and the orientation of individual ZnO NR scatterers based on the distinctive patterns formed at the back focal plane, which are equivalent to Fourier transforming the radiation distribution from the back-aperture of the objective lens. With the ever-growing research and development of single ZnO NRs for highly miniaturized optical, optoelectronic, and biomedical devices, our study’s pursuit of a better understanding of fundamental light interaction with individual 1D ZnO nanomaterials may provide important insight into their potential behavior, particularly when functioning as an effective directional scatterer of polarized light to far field. Furthermore, our efforts may promote innovative optical, hybrid nanophotonic, and biomedical applications based on the use of ZnO NRs by harnessing the polarization-dependent, highly directional optical response of the nanomaterials stemming from their high shape anisotropy.

## 2. Results and Discussion

### Optical Setup for Dark Field Scattering and Back-Aperture Imaging

**Figure 1:**
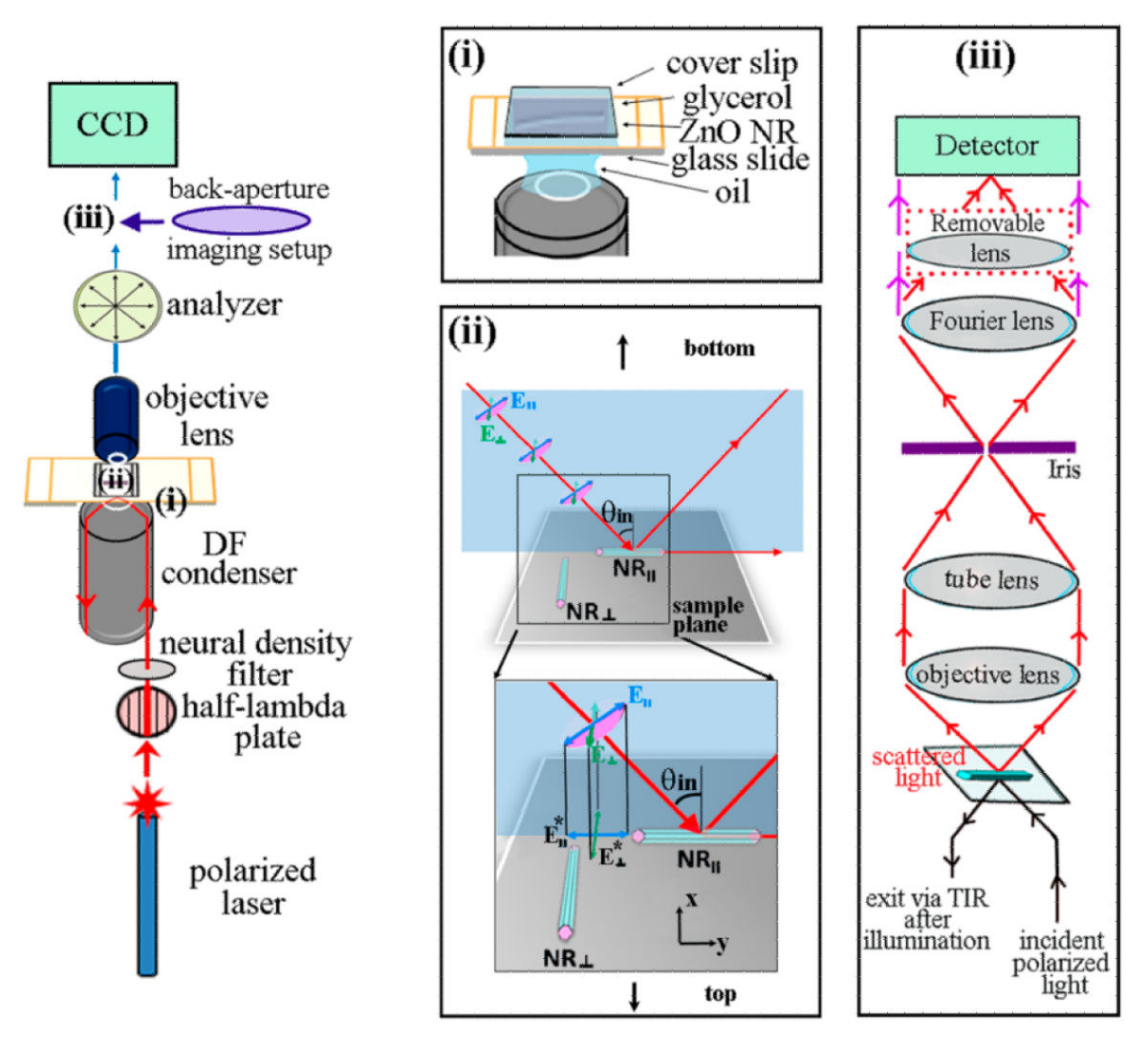
Schematic illustrations showing the experimental setup to measure the NR position- and NR orientation-dependent scattering signal as well as to detect the back-aperture signal from individual ZnO NRs while controlling excitation and collection polarization angles. Three key measurement points in the setup, shown as (i), (ii), and (iii) are displayed in detail inside the boxed panels: (i) sample assembly to achieve refractive index matching for all measurement components and optics, (ii) two distinctive directions for the incoming polarized laser and the two different NR orientations on the measurement plane, and (iii) optical elements to perform forward DF scattering and back-aperture imaging from the same individual NRs. The diagram shown in (ii) is a 180° rotated view of the sample plane and incident light in order to describe the two polarized light directions of **E**_∥_ and **E**_⊥_. The incident angle of the laser, noted as θ_in_ in the diagram, is 62° in our experimental setup. The two orthogonal axes of the sample plane are labelled as x and y. The * sign next to **E** marks the projected components of the electric field onto the sample plane.

Figure 1 describes our experimental setup to measure forward scattering signals from individual ZnO NRs with controlled orientations. The incoming light source, a linearly polarized 642 nm laser, was passed through a half-lambda (HL) plate at 45° and 90° to control the orientation of the incident electric field (**E**) vector to achieve **E**_∥_ (polarization direction lying in the plane of incidence) and **E**_⊥_ (polarization direction perpendicular to the plane of incidence) orientations, respectively. After passing through a series of mirrors and neutral density filters, the laser beam was directed to the sample stage via a dark field (DF) condenser with a high numerical aperture. The use of DF in our setup is essential to resolve the inherently low amount of signal to be collected from individual NR samples. As displayed in panel (i) of Figure 1, multiple components involved in the ZnO NR sample assembly are refractive index-matched throughout all existing interfaces, and this configuration allows for total internal reflection (TIR) of the incident laser beam after illuminating the sample.

The four experimental configurations examined in our forward scattering measurements, displayed in panel (ii) of Figure 1, are the results of different combinations of the polarized laser orientations (**E**_∥_ and **E**_⊥_) illuminating the NRs with the main body lying along the y (NR_∥_) and x (NR_⊥_) directions of the sample plane as defined in the schematics. Additionally, the spatial distribution characteristics of the NR scattering signals are investigated by performing back-aperture imaging using the optical setup provided in panel (iii) of Figure 1. The removal of the last lens between the Fourier lens and the detector in our setup permits back-aperture imaging of the same NR directly after examination of its forward scattering behavior. Using this setup, both forward DF mode scattering and back-aperture images were collected by examining over 20 different individual NRs. The representative scattering characteristics specific to each of the four different combinations of the NR orientation (NR_∥_ and NR_⊥_) and the incident polarization direction (**E**_∥_ and **E**_⊥_) are provided herein.

### Scattering Characteristics of NR_∥_ under E_∥_ and E_⊥_

**Figure 2:**
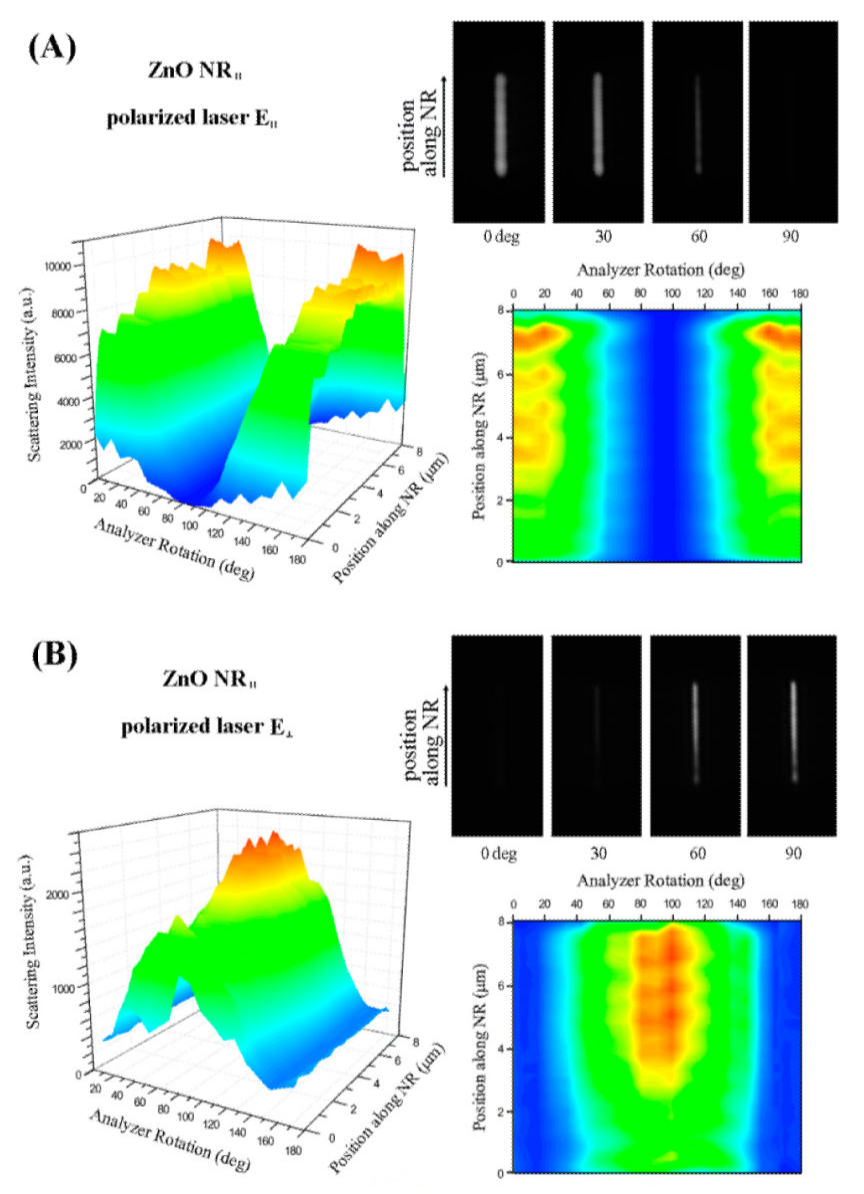
Scattering of a single ZnO NR (138 nm in diameter, 8.06 μm in length) measured by using two polarization directions of an incoming laser (**E**_∥_ and **E**_⊥_) on the NR oriented in the y direction (ZnO NR_∥_**)**. Scattering intensity is measured with respect to the position along the length of the 1D nanomaterial as well as the analyzer angle. (**A**) The 3D contour plot summarizes scattering results from a ZnO NR_∥_ under the excitation of **E**_∥_ as a function of both the analyzer angle and the spatial position on the NR. The highest and lowest scattering is observed when the collection polarization angle is set parallel (0°) and perpendicular (90°) to the incoming polarization direction, respectively. The phenomenon is clearly seen in the 2D projection of the scattering intensity with respect to the analyzer angle at each position along the length of the ZnO NR_∥_. Color schemes used in the 2D plot are the same as the scattering intensity level profiled in the 3D contour graph. A series of grey-scale panels are the scattering images obtained from the ZnO NR measured at the analyzer angle of 0°, 30°, 60°, and 90°, presented from left to right, respectively. (**B**) The same set of scattering measurements was repeated by using the orthogonal excitation of **E**_⊥_ as functions of the analyzer angle and the spatial position on the same ZnO NR_∥_ shown in (A). Similar to what was observed under **E**_∥_, highest and lowest scattering from the NR occurred when the analyzer angle was set parallel and perpendicular to **E**_⊥_, respectively. In (B), those analyzer angles correspond to 90° for the former and 0° for the latter case.

The typical scattering behavior of individual NRs was first characterized from the y axis-oriented NRs (NR_∥_) by employing the two cases of polarized illumination, **E**_∥_ and **E**_⊥_. Figure 2 summarizes the resulting data by showing 3-dimensional (3D) contour plots of the scattering intensity as a function of both the position along the ZnO NR long axis and the analyzer angle, 2-dimensional (2D) projection maps of the scattered signal with respect to the analyzer rotation along the spatial position of the NR, and DF images of the NR_∥_ at four representative analyzer angles of 0°, 30°, 60°, and 90°. Schematics showing the orientations of the key measurement components are also provided in Figure 2. The set of data in Figure 2A represents typical scattering responses of ZnO NRs lying along the y-axis when they are illuminated with an incoming light oriented parallel to the long axis of the NR, *i.e*., **E**_∥_. ZnO NRs used in our study are free of atomic defects and they do not absorb any visible light or show any defect emission in the visible wavelength range. Hence, the scattering signal from individual ZnO NRs collected at the same wavelength as the incident light is not associated with any type of inelastic scattering phenomena. The scattering intensity of the NR_∥_ decreases as the analyzer angle is changed from parallel (0°) to perpendicular (90°) with respect to the incident polarization direction and recovers back to its full scattering intensity when the analyzer rotation is increased from 90° to 180°. This trend in the NR_∥_ scattering intensity is quantitatively confirmed in the 3D contour plot in Figure 2A in which the highest intensities are observed at 0° and 180° while the lowest intensity is observed at 90°. These analyzer angle-dependent changes in NR scattering intensity are further evidenced quantitatively in the 2D projection map and qualitatively in the four representative DF images of the ZnO NR sampled at analyzer rotations of 0°, 30°, 60°, and 90°.

When the same NR_∥_ is examined under the incident laser polarized orthogonal to the NR long axis (**E**_⊥_) instead, analyzer angles at which the maximum and minimum scattering occurs are reversed from the previously discussed **E**_∥_ case. Highest NR_∥_ scattering is observed at the analyzer angle of 90° whilst the lowest is observed at 0° and 180°, as presented in the 3D contour plot in Figure 2B. Similar to the previously discussed case, the 2D projection map of the NR scattering intensity as well as the representative DF panels further demonstrates that, under **E**_⊥_ illumination, the highest (and lowest) scattering from the NR_∥_ is yielded at the analyzer rotation of 90° (and 0° and 180°). For both cases of NR_∥_ scattering using **E**_∥_ and **E**_⊥_, the highest scattering intensity is achieved when the incident **E** field is parallel to the analyzer rotation, while the lowest scattering is observed when the incident **E** field is perpendicular to the analyzer angle. When comparing the measured scattering intensities along the position of the NR at the analyzer angles yielding the maximum signal for each of the **E**_∥_ and **E**_⊥_ case, we notice that the scattering intensity gradually increases from one end towards the other end of the NR. This effect is not likely due to uneven illumination of the NR as the beam size (approximately 50 μm) is much larger than the NR dimensions. Rather, this phenomenon may be caused by the light coupling through the NR end located closer to the incident light wave vector, which is then guided through the NR main body before coupling out through the other end of the NR.

**Figure 3:**
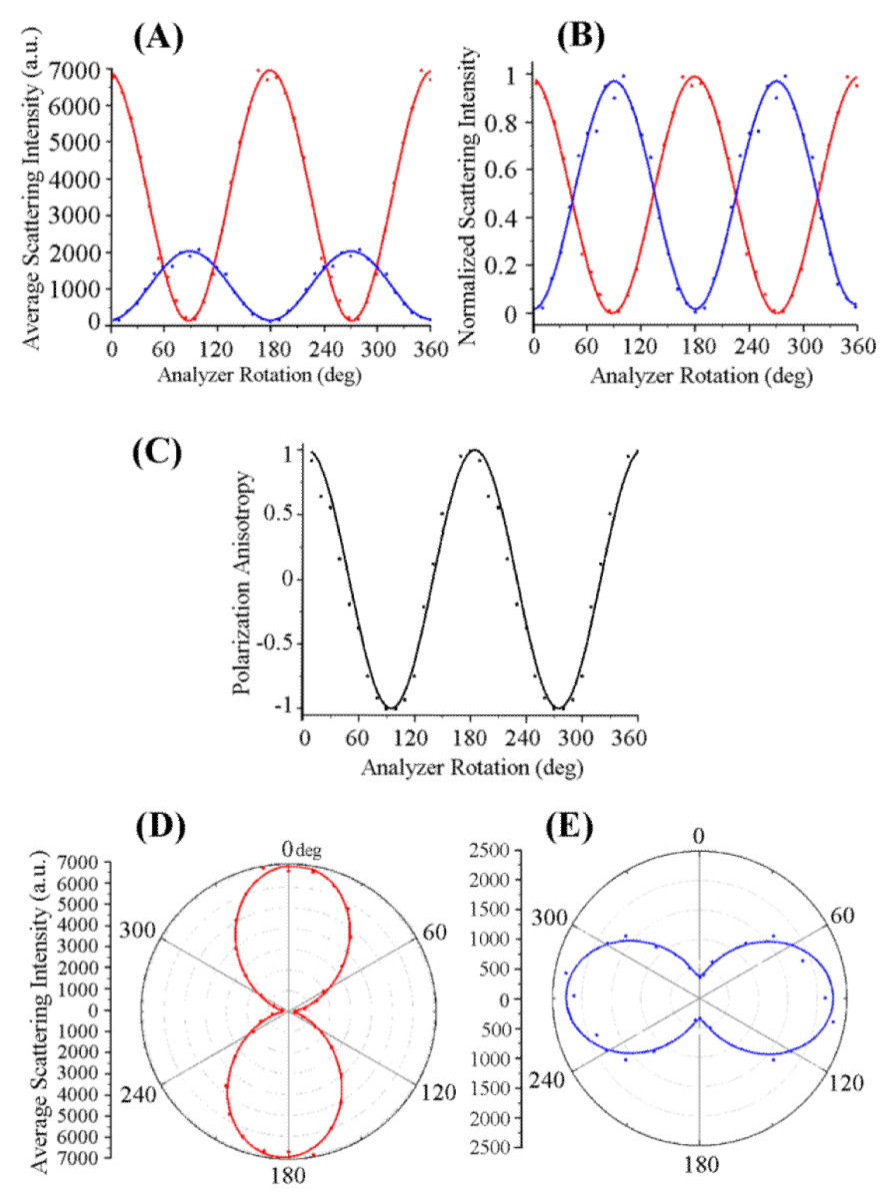
The NR-position dependent scattering signals under the two excitation conditions of **E**_∥_ and **E**_⊥_ were collected over the entire length of the ZnO NR_∥_ and plotted against the analyzer rotation. Red and blue symbols in all graphs are the experimental data when excitation polarizations of **E**_∥_ and **E**_⊥_ were used, respectively. Lines represent curve fits for the corresponding set of data. (**A**) The position-dependent scattering signal was averaged over the entire length of the NR_∥_ and plotted as a function of the analyzer angle. For the same exposure of 50 ms, the overall scattering intensity from the same NR was much lower when **E**_⊥_ was used as excitation instead of **E**_∥_. (**B**) The average scattering intensity was normalized with respect to the highest intensity values measured at each excitation condition and graphed as a function of the analyzer rotation. (**C**) Polarization anisotropy values calculated from the data shown in (B) follow a cos^2^θ dependence on the analyzer angle, θ. (**D,E**) Polar plots of average scattering intensities from the ZnO NR_∥_ under (D) **E**_∥_ and (E) **E**_⊥_ excitation are shown.

In Figure 3, the scattering signal over the entire length of the ZnO NR_∥_ is further processed and compared for the two excitation conditions of **E**_∥_ and **E**_⊥_ by plotting NR-position averaged scattering values against the analyzer rotation. Specifically, the scattering dependence of the ZnO NR_∥_ on the incoming **E**-field polarization is evaluated by plotting the average scattering intensity, normalized scattering intensity, and polarization anisotropy (PA) as a function of the analyzer rotation in Figure 3A, B, and C, respectively. In all graphs of Figure 3, data collected from the two cases of **E**_∥_ and **E**⊥ (and their respective curve fits) are represented as red and blue points (and lines), respectively. For the normalized intensity plots, the average scattering intensity is normalized with respect to the highest and lowest intensity values measured at each excitation condition. The PA values plotted in Figure 3C are obtained by the equation shown below, which defines PA as the ratio of the difference between the scattered light intensities for polarized light under **E**_∥_ and **E**_⊥_ to the sum of those values at a given analyzer angle.

(1) $$\mathrm{PA}=\frac{I_{E\|}-I_{E^{⊥}}}{I_{E\|}+I_{E^{⊥}}}$$

When the scattering intensity values are taken at the analyzer angles allowing the highest signals for **E**_∥_ and **E**_⊥_ (**I** of 6800 and 2000, respectively), then the above equation yields a PA value of 0.55 from this measurement configuration.

We note a characteristic trend of the NR_∥_ scattering intensity (**I**) as function of analyzer angle, which reveals a sinusoidal pattern as shown in Figure 3 A, B, and C. When fitting the experimental data obtained under the two cases of **E**_∥_ and **E**_⊥_, the scattered signal from the ZnO NR_∥_ is directly proportional to the square of the cosine of the angle (θ) between the transmission axis of the analyzer and the incident polarized light. This behavior is similar to what is known as Malus’s law, which describes the angle-dependent intensity of plane-polarized light incident on an analyzer and demonstrates that the same macroscopic behavior can be carried over to explain the analyzer angle-dependent intensity of a scatter whose width is only a few hundred nanometers. In these instances, the intensity of the light transmitted by the analyzer is directly proportional to the square of the cosine of the angle between the transmission axes of the analyzer and the polarizer, **I** = **I_0_** cos^2^(θ). Furthermore, in comparing the average intensity values of the scattered light from the same ZnO NR_∥_ between **E**_∥_ and **E**_⊥_, the maximum NR scattering intensity under **E**_∥_ is approximately 3.5 times greater than scattering from **E**_⊥_. This value was obtained by keeping the exposure time constant at 50 ms for both orientations of the incident light. Figure 3D,E provides polar plots of the ZnO NR_∥_ in order to reveal the effect of **E**_∥_ and **E**_⊥_ on the degree of polarization in ZnO NR_∥_. When evaluating the corresponding polar intensity plots in Figure 3D,E for the effect of **E**_∥_ and **E**_⊥_ on NR_∥_ scattering, a stronger polarization anisotropy effect is seen under **E**_∥_ as evidenced by the narrower waist of the dipolar plot in Figure 3D in comparison to that in Figure 3E. NRs with high shape anisotropy will result in dipolar scattering patterns with tightly closed centers, whereas the patterns will open up in the center and become circular instead when the length of the nanomaterial reaches its width.

### Scattering Characteristics of NR_⊥_ under E_∥_ and E_⊥_

**Figure 4:**
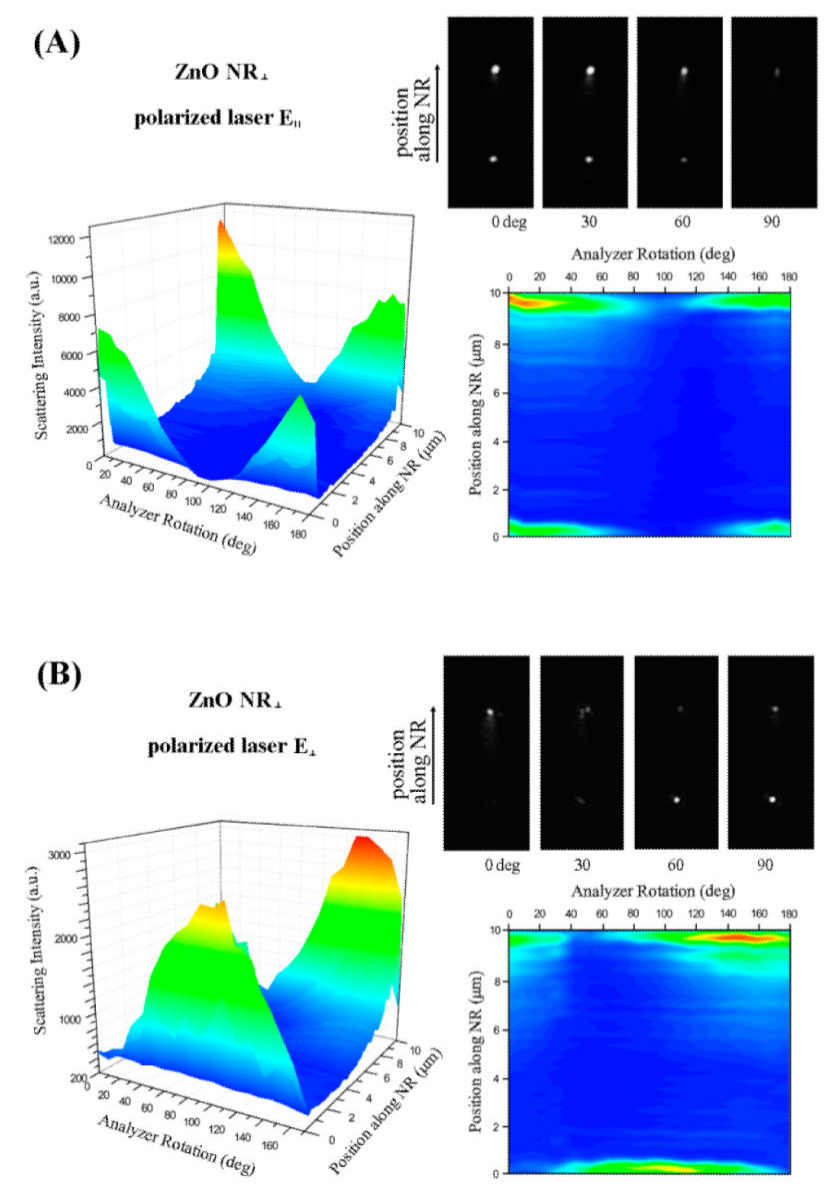
Scattering of a single ZnO NR (184 nm in diameter, 10.15 μm in length) measured by using two polarization directions of an incoming laser (**E**_∥_ and **E**_⊥_) on an NR oriented along the x-axis (ZnO NR_⊥_). Scattering intensity was measured with respect to the position along the length of the 1D nanomaterial as well as the analyzer angle. (**A**) The 3D contour plot summarizes scattering results from a ZnO NR_⊥_ under the excitation of **E**_∥_ as a function of both the analyzer angle and the spatial position on the NR. A striking difference in the scattering signal was seen along the length of the NR_⊥_. Intense scattering occurred only on the two ends of the NR_⊥_ where the signal along the main body of NR_⊥_ was negligible. This phenomenon is also clearly seen in the 2D projection of the scattering intensity with respect to the analyzer angle at each position along the length of the ZnO NR_⊥_. Color schemes used in the 2D plot are the same as the scattering intensity levels profiled in the 3D contour graph. A series of grey-scale panels are the scattering images obtained from the ZnO NR measured at the analyzer angle of 0°, 30°, 60°, and 90°, respectively. (**B**) The same set of scattering measurements was repeated by using the orthogonal excitation of **E**_⊥_ as a function of both the analyzer angle and the spatial position on the same ZnO NR_⊥_ shown in (A).

Subsequently, the scattering characteristics of x-axis oriented ZnO NRs (NR_⊥_) were investigated as a function of the position along the NR_⊥_ and the analyzer rotation for both the incident **E** fields of **E**_∥_ and **E**_⊥_. The results are provided in Figure 4 along with the schematics showing the orientations of the key measurement components. The 2D projection maps and 3D contour plots in Figure 4A,B display the typical scattering response of NR_⊥_ under the incident light with **E**_∥_ and **E**_⊥_ polarizations, respectively, as a function of the analyzer angle.

The data in Figure 4 reveal that the scattering behavior of NR_⊥_ is strikingly different than that of NR_∥_. The scattering signal from the NR_⊥_ is highly localized at the two ends (basal planes) of the NR_⊥_ upon illumination with either **E**_∥_ or **E**_⊥_. Variations in the analyzer rotation lead to changes in this highly localized scattering intensity at each end, resulting in either even brightening/dimming of the two ends under **E**_∥_ or alternating brightness between the two ends under **E**_⊥_. This tendency is displayed in the four DF panels provided in Figure 4A,B. At the same time, scattering is completely absent along the main body (prismic planes) of the NR_⊥_ despite the variations in **E**_∥_ and **E**_⊥_ or in the analyzer setting. This discontinuity in scattering intensity along the position of the NR_⊥_ is embodied as the NR edge peaks in the 2D and 3D plots of Figure 4 as well as in the one (or two) bright NR end spots in the four representative DF images. In comparison, the scattering intensity from NR_∥_ detailed in Figures 2 and 3 is relatively uniform spatially along the length of the NR_∥_, regardless of the polarization of the incident light.

Both the 2D projection maps and 3D contour plots in Figure 4A,B clearly present the spatially resolved, NR_⊥_ scattering intensity along the position on the NR as a function of the analyzer angle probed from 0° to 180° with an increment of 10°. When the analyzer setting is varied incrementally from 0° to 90° under **E**_∥_, a decreasing trend in NR_⊥_ scattering intensity is observed as seen in the DF panels in Figure 4A. The data in Figure 4A display the highest intensities monitored at 0° and 180° while the lowest is observed at 90°, showing the same trend as those observed in the (NR_∥_, **E**_∥_) case. This observation is due to the maximum scattering intensity being transmitted through the analyzer when its polarization vector is aligned with the polarized direction of the incident light. On the other hand, the scattering intensity of the same NR_⊥_ under **E**_⊥_ in Figure 4B shows more complicated behaviors. In this case, a small degree of polarization demixing is observed and the scattering does not become uniformly weaker at all positions of the NR even at the analyzer angle perpendicular to the incident polarization. Although the cause is not understood yet, this effect leads to the an interesting optical phenomenon evidenced in Figure 4B by the switching of the bright ends in the series of NR scattering panels at different analyzer rotation, as well as in the 2D map showing the alternating analyzer angles of the two NR ends corresponding to the maximum scattering intensity. The observed rotation of the polarization from one end to the other of the NR may be related to the fact that ZnO is a birefringent material.

**Figure 5:**
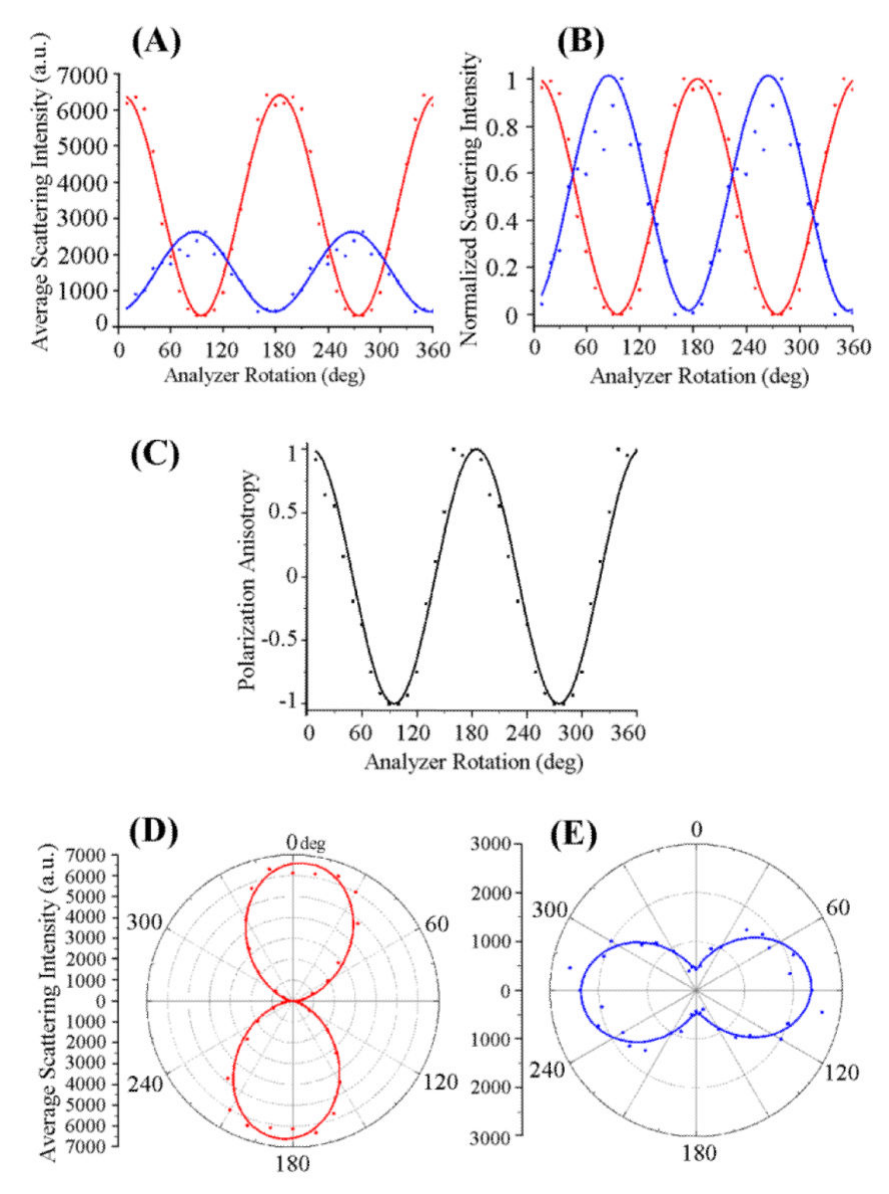
The NR position-dependent scattering signals under the two excitation conditions of **E**_∥_ and **E**_⊥_ are collected over the entire length of the ZnO NR_⊥_ and plotted against the analyzer rotation. Red and blue symbols in all graphs are experimental data when the excitation polarizations of **E**_∥_ and **E**_⊥_ are used, respectively. Lines represent curves fits for the corresponding set of data. (**A**) The position dependent scattering signal averaged over the entire length of the NR_⊥_ is plotted as a function of the analyzer angle. The overall scattering intensity from the same NR was much lower when **E**_⊥_ was used as excitation instead of **E**_∥_ while keeping the same exposure time of 10 ms. (**B**) The average scattering intensity was normalized with respect to the highest intensity values measured at each excitation condition and graphed as a function of analyzer rotation. (**C**) Polarization anisotropy values calculated from the data shown in (B) follow a cos^2^θ dependence on the analyzer angle, θ. (**D and E**) Polar plots of average scattering intensities of the ZnO NR_⊥_ under (D) **E**_∥_ and (E) **E**_⊥_ excitation are displayed.

In order to substantiate the polarization-dependent scattering behavior of ZnO NR_⊥_ under the two cases of the incident laser, Figure 5 further displays the quantitative scattering data measured from the ZnO NR_⊥_ discussed in Figure 4. Figure 5A displays the scattering signal averaged over the entire length of the NR_⊥_ in response to **E**_∥_ (red points) and **E**_⊥_ (blue points) while systematically varying the analyzer angle. Red and blue lines in the graphs are the curve fits of the respective data. Figure 5B shows the scattering intensity as a function of analyzer rotation after normalizing the signals with respect to the highest and lowest intensity values measured at each excitation condition. The exposure time was kept constant at 10 ms between the two laser polarizations. The scattering intensity of the NR_⊥_ is approximately 2.5 times greater under **E**_∥_ irradiation than under **E**_⊥_, yielding a PA value of 0.428. When taking the different NR orientations into consideration, a larger difference between the average scattering intensity values from **E**_∥_ and **E**_⊥_ illumination is observed for NR_∥_ than for NR_⊥_. Figure 5C presents the calculated PA values at each analyzer angle for the NR_⊥_. Figure 5D,E provide polar intensity plots of the average scattering intensities from the ZnO NR_⊥_ under **E**_∥_ (red) and **E**_⊥_ (blue) radiation. Similar to the behavior observed in NR_∥_, the polar plots show a dipole-like pattern with a tightly closed center for **E**_∥_ excitation while the dipolar plot is slightly open at the center under **E**_⊥_.

### Scattering Behavior Distinctive to Each Light-Matter Pair of (NR_∥_, E_∥_), (NR_⊥_, E_⊥_), (NR_∥_, E_⊥_) and (NR_⊥_, E_∥_)

The most striking difference in scattering behaviors for the four cases shown in Figures 2 and 4 can be summarized by the fact that y- and x-axis oriented ZnO NRs give rise to continuous (relatively evenly distributed) and discontinuous (highly localized) scattering, respectively, when analyzed as a function of the position on the NR. As detailed above, the scattering signal is observed only from the end(s) of NR_⊥_ regardless of the orientation of the incident polarized light and the analyzer rotation, whereas scattering signal from NR_∥_ is present continuously from all positions of the NR. If the incident light enters normal to the sample plane, the symmetry in measurement conditions between the NR long axis and polarization direction present in the set of (NR_∥_, **E**_∥_) and (NR_⊥_, **E**_⊥_) as well as in the set of (NR_∥_, **E**_⊥_) and (NR_⊥_, **E**_∥_) will yield identical scattering patterns for the two cases within the same set. Our data, summarized in Figures 2 and 4 and displaying distinctively different scattering behaviors monitored from the four different cases, correspond to the glancing laser light entering with an incident angle (θ_in_) of approximately 62°, as illustrated in Figure 1(ii). Further work is currently underway via computer simulation studies to understand the exact roles of the glancing incident light and the possible origins of the distinctive scattering profiles specific to each polarization and NR orientation case.

### Back-Aperture Imaging

**Figure 6:**
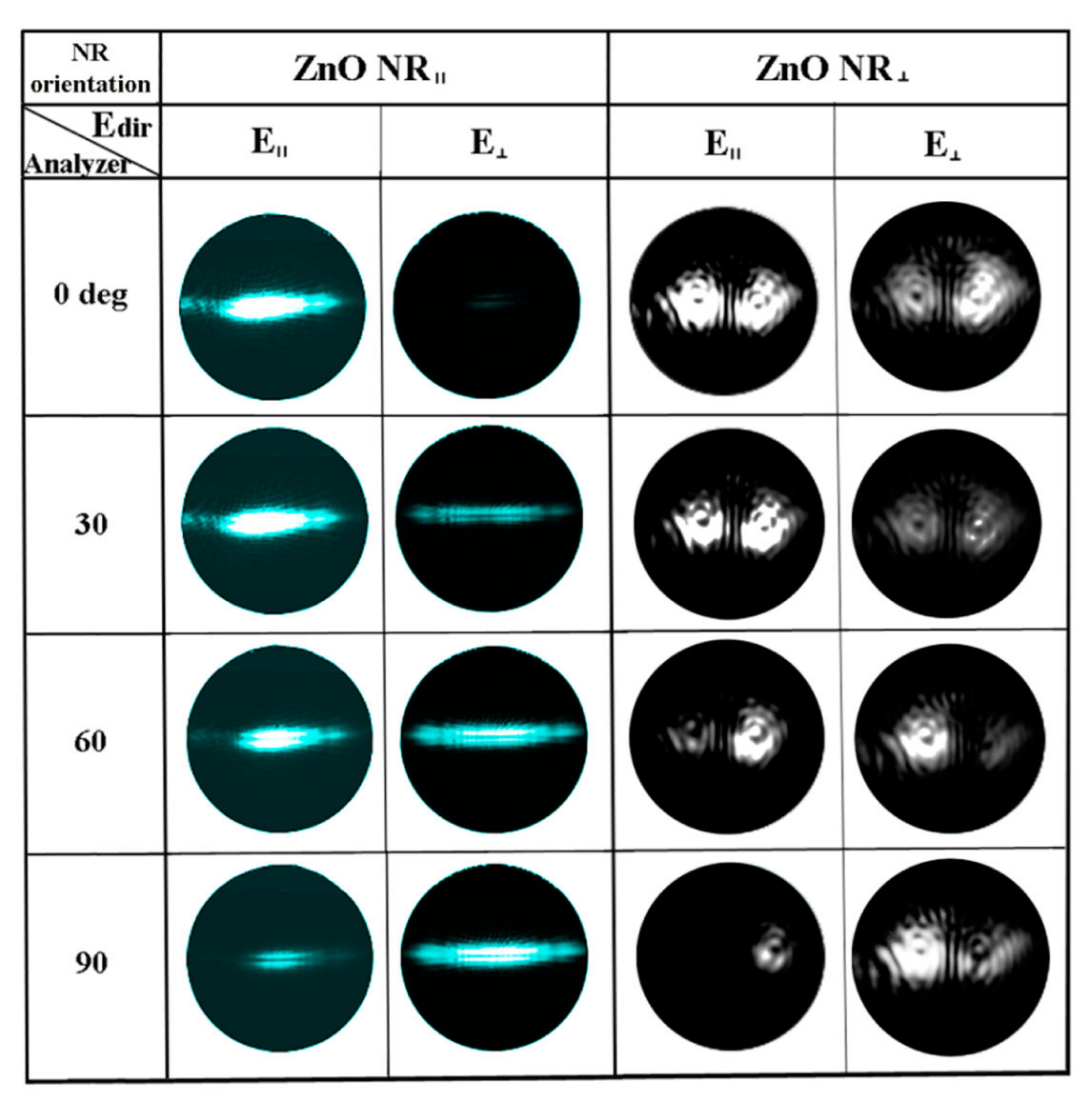
Back-aperture images of the scattering signal from ZnO NRs of different spatial orientations when varying the polarization direction of excitation as well as the analyzer angle. To collect the set of back-aperture images, the excitation direction of **E**_∥_ and **E**_⊥_ were used at representative collection angles of 0°, 30°, 60°, and 90°. ZnO NR_∥_ exhibited a linear radiation pattern rotated 90° from the physical orientation of the NR in the measurement plane. ZnO NR_⊥_ displayed ripple-like radiation patterns emanating from two focal points (the two scattering ends of the NR_⊥_), that mimic interference patterns seen from radially propagating waves from two different centers.

We subsequently examined the polarization-dependent spatial distributions of the scattering patterns from individual ZnO NRs by carrying out back-aperture imaging as shown in the schematics of Figure 6. Superpositioned plane waves, each defined by a unique wave vector in reciprocal space, radiate from ZnO NRs, and their directional and spatial scattering information can be accessed by back-aperture imaging. This is possible since the objective lens focuses these individual plane waves into different spots with unique spatial coordinates at the back focal plane of the lens. Hence, the back focal plane of the objective lens contains the spatial information on scattering directionality. Back-aperture imaging refers to acquiring Fourier transformed images of the scattering electric field distribution from the objective lens on the back focal plane, a plane perpendicular to the optical axis of the objective lens at its focal distance. For back-aperture imaging of the NRs, a set of optical elements containing a Fourier lens was used as displayed in the setup in Figure 1(iii). This setup enabled us to carry out the back-aperture imaging of the same NRs immediately after the forward scattering measurements, while collecting the signal only from the NR of interest with the use of an iris. To switch back and forth between forward scattering and back-aperture imaging modes, the removable lens, which is the last lens between the iris and the detector, is simply inserted into the imaging path to form a focused image on the detector for the forward DF scattering and removed to collimate the beam for the back-aperture imaging, as indicated with the beam path in red and purple in Figure 1(iii), respectively. Similar to the forward scattering experiment, both directionality and intensity of the scattered radiation from individual NRs are probed with respect to the angle between analyzer and the transmission axis.

Specifically, we carried out back-aperture imaging for both NR_∥_ and NR_⊥_ under **E**_∥_ and **E**_⊥_ illumination at the analyzer setting from 0° to 170° for measurements at every 10°. The resulting scattering patterns at four representative analyzer angles of 0°, 30°, 60°, and 90° are presented in Figure 6A and categorized by the polarization direction of the incident **E** vector (**E**_dir_), the analyzer angle (θ), and the orientation of the NR. For the ZnO NR_∥_ in both the cases of **E**_∥_ and **E**_⊥_, the back-aperture images reveal that the scattering patterns appear as a strip of horizontal bands that are perpendicular to the NR orientation on the back focal plane. For the NR_∥_, the scattering intensity is the strongest when the incident **E** field is parallel with the analyzer rotation at 0° for **E**_∥_ and 90° for **E**_⊥_, while the emission is weakest when the **E** field is perpendicular to the analyzer at 90° for **E**_∥_ and 0° for **E**_⊥_. This tendency agrees with the forward scattering intensity of ZnO NRs whose results were discussed earlier. For ZnO NR_⊥_, a ripple-like scattering pattern is observed instead for both **E**_∥_ and **E**_⊥_ illumination. The rippled patterns are formed due to the interference patterns of the concentrically propagating waves originating from the two points that correspond to the two end scattering points of the NR_⊥_. Although the intensity of the NR_⊥_ back focal pattern is most pronounced when the incident **E** field is parallel with respect to the analyzer angle at 0° for **E**_∥_ and at 90° for **E**_⊥_, the intensity differences are not as noticeable as for the case of NR_∥_. In our forward scattering measurements on NR_∥_ described in Figures 2 and 3, the entire NR scatters light along the entire NR length. This position-independent presence of scattering signal results in the linear band-like pattern recorded on the back focal imaging plane. Light response from NR_⊥_ in our forward scattering measurements is observed only at the two end facets of the NR as discussed in Figures 4 and 5, and the presence of its scattering signal is highly sensitive to the position on the NR. This effect is manifested into the ripple-like patterns on the back-aperture. Therefore, our back focal imaging results of ZnO NRs confirm the optical response observed in our forward scattering measurements.

The polarization-dependent scattering response resolved in this study can, therefore, provide insight into the optical signal expected from individual ZnO NRs depending on their orientation in the measurement plane. Highly increased signal confined in the NR termini has been previously reported in near band edge (NBE) photoluminescence and biomolecular fluorescence of ZnO NRs, although the effect of polarization-, orientation-, and position-dependence of the NRs was not systematically evaluated. When unpolarized light was used as an excitation source to probe NBE emission from ZnO NRs, the two NR ends exhibited stronger photoluminescence intensity when compared to the NR body [22]. In addition, an interesting phenomenon of fluorescence intensification on ZnO NR ends was reported in recent studies involving fluorophore-coupled biomolecules on individual ZnO NRs [36,39]. The outcomes of our study demonstrate that a similar, highly spatially localized scattering can be achieved by controlling the NR orientation with respect to the direction of light polarization. Therefore, our results from this study suggest that even richer and novel optical behaviors can be identified when scattering, photoluminescence, and fluorescence/Raman emission of individual nanomaterials are further examined with well-characterized light polarization and nanomaterial orientation. Our efforts, signifying a systematic scattering investigation of the non-trivial case of glancing incident light with controlled polarization, may be highly valuable in interpreting and predicting characteristic optical responses collected from individual NRs of specific orientations. Coupled with the capability of discerning scattering intensity along the position of the NR as well as elucidating the spatial distribution characteristics of the scattered light, our endeavors may be extremely beneficial to the optimal design of optical devices with improved sensitivity and advantageous in terms of accurate interpretation of the collected optical signal based on the light polarization direction and NR orientation. We also note that further work pertaining to the intriguing ZnO NR scattering profiles is ongoing by examining the system with different wavelengths and NR orientations in order to elucidate the fundamental mechanisms leading to the NR orientation-dependent scattering patterns as well as the spatial distributions.

## 3. Summary

In summary, we have performed forward scattering measurements of single ZnO NRs in a dark-field configuration and elucidated their characteristic, polarization-resolved optical response under well-controlled experimental parameters such as incident light polarization, NR orientation, and analyzer rotation. We have also carried out back-aperture imaging of the same individual ZnO NRs and systematically examined the spatial radiation directivity of the scattering signal. We have quantitatively catalogued the effect of light polarization, NR orientation, and position along the NR on both the intensity of the resulting scattering signal as well as the corresponding spatial distribution pattern of ZnO NR scattering. Since fundamental light interaction behavior of NRs will significantly affect their functional outcomes in photonics, optoelectronics, and sensor devices, our endeavors presented in this paper will not only provide much needed insight into the unique optical properties of individual 1D ZnO nanomaterials in light-matter interaction but also signify an important step forward to developing next-generation optoelectronic systems and optical biodetectors with improved device efficiency and sensitivity.

## 4. Materials and Methods

### Sample Growth and Assembly

1 × 1 cm Si wafer (0.017 in. thickness) obtained from Silicon, Inc was cleaned by sonication in ethanol and dried under a stream of N_2_. For individual ZnO NRs, 20 μL of 20 nm Au colloid (Ted Pella, Inc) was deposited on a Si wafer for 5 min and gently blown dry with N2. The source materials, 0.45 grams of a 2:1 mixture of graphite (99%) and zinc oxide powders (99.999%) obtained from Alfa Aesar Inc., were placed in a quartz boat at the center of a horizontal resistance furnace, and a target boat containing the catalyst-deposited Si substrate was placed 15.6 cm downstream. The furnace was heated to 950 °C for 2 h at a ramp up/ramp down rate of 15°C/min under a constant Ar flow of 100 standard cubic centimeters per minute. Various aspect ratios of ZnO NRs were achieved by slight alterations to the distance between the source materials and the growth substrate. ZnO NRs were subsequently sonicated off their growth substrates in ethanol and 2 μL aliquots of dispersed ZnO NRs were subsequently drop-casted onto on to a clean glass slide and then dried with N_2_. A small drop of glycerol (n = 1.4729) was placed on top of the ZnO NRs on the glass slide and a cover slip was slowly lowered onto the sample while avoiding bubble formation.

### Forward Dark-Field Scattering and Back-Aperture Imaging

When performing the forward scattering measurements, a linearly polarized 642 nm laser (Spectra Physics Excelsior-PS-DD-CDRH with an average output power of 60 mW, Newport Corp., Bozeman, MT) was focused onto the ZnO NR plane from below the sample stage after passing through a half-lambda (HL) wave plate and a neutral density filter wheel (Thorlabs Inc., Newton, NJ). The HL plate controlled the orientation of the **E** field vector of the laser on the NR plane, as seen in Figure 1. The polarized laser was directed to the sample through a high numerical aperture (N.A. = 1.2~1.4), oil-immersion DF condenser mounted below the stage and subsequently focused onto individual NRs of investigation. An analyzer was placed between the microscope tube lens and the CCD detector to examine polarization anisotropy of the scattered light from individual NRs. Scattering signal was collected by using a 40 × plan apochromatic objective lens (Olympus PlanSApo, N.A. = 0.90, Olympus Corp., Center Valley, PA). Scattered light from the ZnO NR sample was collected at specific analyzer angles in order to investigate the polarization dependence. For a given NR, a set of eighteen analyzer settings were used from 0° to 170° in 10° interval. Additionally, back-aperture imaging of individual ZnO NRs was performed by adding a home-built optical attachment to our forward scattering setup. The optical addition employed to examine the spatial distribution of the scattered light contained an iris and a removable lens. Data from the forward scattering and back-aperture imaging measurements were processed and analyzed using Origin 8.5 software (OriginLab Corp., Northampton, MA) and Image J, the Java-based image-processing program.
